# An Integrated Pan-Cancer Analysis of 33 Human Cancers Reveals the Potential Clinical Implications and Immunotherapeutic Value of C-X-C Motif Chemokine Ligand 13

**DOI:** 10.3389/fonc.2022.791962

**Published:** 2022-01-24

**Authors:** Huanyu Zhang, Honghao Yin, Jing Chen, Yuan Yuan

**Affiliations:** ^1^ Tumor Etiology and Screening Department of Cancer Institute and General Surgery, The First Hospital of China Medical University, Shenyang, China; ^2^ Key Laboratory of Cancer Etiology and Prevention in Liaoning Education Department, The First Hospital of China Medical University, Shenyang, China; ^3^ Key Laboratory of GI Cancer Etiology and Prevention in Liaoning Province, The First Hospital of China Medical University, Shenyang, China

**Keywords:** CXCL13, pan-cancer, prognosis, immune cell infiltration, immune checkpoint, immunotherapy

## Abstract

**Background:**

C-X-C Motif Chemokine Ligand 13 (CXCL13) plays a crucial part in the pathogenesis of numerous lymphoproliferative disorders, inflammatory responses, and autoimmune diseases. CXCL13 also influence tumor development and prognosis, and be a potential target for cancer treatment. However, CXCL13 expression-based panoramic picture in pan-cancer remain unclear. This study focused on elucidating different expression levels, prognostic significance, immune-related characteristics, epigenetic variations, and immunotherapeutic value of CXCL13.

**Methods:**

Based on different databases such as TCGA, GTEX, CCLE and HPA, we studied the expression of CXCL13 in different tissues at different levels. Moreover, we analyzed the correlation between CXCL13 expression and clinicopathological characteristics, prognosis, Mismatch Repair Genes (MMRs), Microsatellite Instability (MSI), tumor mutation burden (TMB), immune cells infiltration, immune-related genes, and the role in tumor immunotherapy. And the expression of CXCL13 in digestive tract cancers and the correlation between CXCL13 and immune genes were further analyzed by histological verification.

**Results:**

CXCL13 was highly expressed in various tumor tissues and was also closely related to prognosis. CXCL13 expression levels were closely related to MSI, TMB and immune cells infiltration, including CD8+ T cells, macrophages, follicular helper T cells and B cells. CXCL13 expression levels were related to immune checkpoint genes and the efficacy of immunotherapy.

**Conclusion:**

CXCL13 might be a useful biomarker for determining the diagnosis and prognosis of human cancers but also a biomarker for evaluating the efficacy of immunotherapy.

## Introduction

The burden of cancer morbidity and mortality is growing rapidly worldwide, and cancer is recognized as the leading cause of death and a major barrier to life extension in every country globally. The first line of treatment for most patients is conventional, including surgery, chemotherapy and radiotherapy. Recently, accumulating research has indicated that immunotherapy has become the focus trend in cancer treatment and Immune checkpoint inhibitors (ICIs) are eutherapeutic in some cancers. However, there are significant differences in the efficacy of immunotherapy among different cancer types or patients with the same cancer type. In recent years, clinical and basic studies have contributed increasing potential biomarkers to evaluate the efficacy of immunotherapy, but there are still many problems, such as the lack of systematic evaluation of biomarkers, the internal relationship between them and the molecular mechanism of biomarkers also needs to be further investigated.

C-X-C Motif Chemokine Ligand 13 (CXCL13) belongs to the chemokine family and is also known as B lymphocyte chemoattractant or B cell-attracting chemokine-1, which is one of the main chemokines involved in the formation of tertiary lymphoid structures (TLSs) (1–5). CXCL13 plays a crucial part in infectious, inflammatory and immune responses and is closely related to the pathogenesis of numerous lymphoproliferative disorders, inflammatory and autoimmune diseases (6). In recent years, CXCL13 has also been found to be closely associated with tumorigenesis and may directly or indirectly modulate the migration and proliferation of tumor cells, influence tumor development and prognosis, and be a potential target for cancer treatment (7). CXCL13+BHLHE40+ Th1-like cells are potential markers of colorectal cancer immunotherapy (8) and CXCL13 was suggested as a predictive biomarker for ICI response in bladder cancer (9).

To date, research on CXCL13 in cancers has been limited to a specific type of cancer, so it is necessary to perform a pan-cancer analysis on the target gene to evaluate its interrelationship with underlying molecular mechanisms, clinical phenotypic characteristics and tumor immune microenvironment. Genome-wide pan-cancer analysis aids in understanding the common events in the occurrence and development of cancer and can help design cancer prevention, diagnosis, and treatment strategies. There are different public databases available containing functional genomics datasets for various cancers, which can be utilized for this analysis. Moreover, the expression of CXCL13 in digestive tract cancers were further analyzed by histological verification. The present study was the first to use multi-level data from different databases to perform a CXCL13 expression-based pan-cancer analysis and the results indicate that CXCL13 can be used as a risk or prognostic factor for many cancers, and it plays an important role in tumor immunity by affecting TMB, MSI and tumor infiltrating immune cells. It can provide a basis for in-depth understanding of the role of CXCL13 in tumor immunotherapy.

## Materials And Methods

### Data Collection and Standardization Criteria

Our study extracted CXCL13 mRNA expression data of 33 different tumor types from TCGA (The Cancer Genome Atlas) and 31 normal human tissues (7858 samples) from the Genotype-Tissue Expression (GTEx) using UCSC XENA (https://xenab.rowser.net/). TCGA data were analyzed in conjunction with clinical data (e.g., survival time, grade, stage, and survival status). Further, log2 transformation was performed for each expression value. Finally, cancer species with less than 3 samples in a single cancer species were removed, and abbreviations for all types of cancer were on display, as shown in **Table 1**. Cellular CXCL13 data for 24 different kinds of cell lines were obtained from the Broad Institute Cancer Cell Line Encyclopedia (CCLE) portal (https://portals.broadinstitute.org/ccle/about).

**Table 1:** 33 types of human cancers employed in our research.

Abbreviation	Full name
ACC	Adrenocortical carcinoma
BLCA	Bladder urothelial carcinoma
BRCA	Breast invasive carcinoma
CESC	Cervical squamous cell carcinoma and endocervical adenocarcinoma
CHOL	Cholangiocarcinoma
COAD	Colon adenocarcinoma
DLBC	Lymphoid neoplasm diffuse large B-cell lymphoma
ESCA	Esophageal carcinoma
GBM	Glioblastoma multiforme
HNSC	Head and neck squamous cell carcinoma
KICH	Kidney chromophobe
KIRC	Kidney renal clear cell carcinoma
KIRP	Kidney renal papillary cell carcinoma
LAML	Acute myeloid leukemia
LGG	Brain lower grade glioma
LIHC	Liver hepatocellular carcinoma
LUAD	Lung adenocarcinoma
LUSC	Lung squamous cell carcinoma
MESO	Mesothelioma
OV	Ovarian serous cystadenocarcinoma
PAAD	Pancreatic adenocarcinoma
PCPG	Pheochromocytoma and paraganglioma
PRAD	Prostate adenocarcinoma
READ	Rectum adenocarcinoma
SARC	Sarcoma
SKCM	Skin cutaneous melanoma
STAD	Stomach adenocarcinoma
TGCT	Testicular germ cell tumors
THCA	Thyroid carcinoma
THYM	Thymoma
UCEC	Uterine corpus endometrial carcinoma
UCS	Uterine carcinosarcoma
UVM	Uveal melanoma

### Analysis of CXCL13 Expression

The CXCL13 expression status in 31 normal human tissues and 24 cell lines were analyzed using the Kruskal-Wallis test. p < 0.05 was considered statistically significant. Differential CXCL13 expression between normal and tumor tissue was determined by the wilcox-test. A False Discovery Rate (FDR) value < 0.05 was used as a cut-off. FDR < 0.05, < 0.01, < 0.001 were represented by *, **, *** respectively.

### Immunohistochemical Staining

We utilized The Protein Atlas dataset (https://www.proteinatlas.org/) to gather the immunohistochemical staining images of CXCL13 including 4 types of tumor tissues (UCEC, TGCT, OV, PRAD) and their corresponding Normal tissues to evaluate the protein-based differences of CXCL13. We downloaded histological section images and corresponding information of CXCL13 from cancer and normal tissues obtained by immunohistochemistry in HPA. The expression of CXCL13 was evaluated by staining intensity and percentage of staining, respectively: intensity was given scores 0–3 (0 = negative, 1 = weak, 2 = moderate, 3 = strong), and the percentage of immunopositive cells was given scores 0–3 (0 = 0%, 1 = 1%-25%, 2 = 26-75%, 3 = 76%-100%). IHCscore = (score of intensity) × (score of percentage of staining). Then, Mann-Whitney U test was performed with SPSS 20.0 software to compare CXCL13 staining levels between cancer tissues and normal tissues. The cut-off P value was set to 0.05.

### Correlation Analysis for CXCL13 Expression Levels and Clinicopathological Characteristics or Survival in Pan-Cancer

We investigated the correlation of the expression of CXCL13 and clinicopathological characteristics (age, gender and pathological stages). Furthermore, Kaplan-Meier method and univariate cox regression analysis were used to illustrate the correlations between the CXCL13 expression levels and patient prognosis in 33 cancer types. The following four terms for patient prognosis were included: overall survival (OS), disease-specific survival (DSS), progression-free interval (PFI), and disease-free interval (DFI). Univariate survival analysis was used to calculate the hazard ratio (HR) and 95% confidence intervals. Meanwhile, we tested the correlation between clinical outcomes (age, gender and tumor stages) and survival using Log-rank test. P values were adjusted using the Benjamini-Hochberg procedure for pairwise comparison of clinical stages. Adjusted P-value < 0.05 were considered significant.

### Correlation Analysis for CXCL13 Expression Levels and MMRs, MSI, and TMB in Pan-Cancer

The expression data for five Mismatch Repair Genes (MMRs) (EPCAM, PMS2, MSH6, MSH2, and MLH1) and the Microsatellite Instability (MSI) scores were acquired from the TCGA database. The number of mutations in each tumor sample was calculated and then divided by the total exon length. The corrected number of mutant bases per million bases was defined as the tumor mutation burden (TMB). The associations between CXCL13 expression levels and MMR gene expression levels, MSI or TMB was evaluated by Spearman’s method. The cut-off value was |r | ≥ 0.2 and p < 0.05. RStudio 3.6.3 was used for data analysis.

### Association Between CXCL13 Expression and Immune-Related Factors in Pan-Cancer

The stromal and immune scores were calculated in pan-cancer tissues with the ESTIMATE algorithm (10) to infer the degree of stromal or immune cells infiltrating the tumor by R-package “estimate”. Data for immune-related genes (chemokine, chemokine receptors, MHC, immunosuppressive genes, immunoactivating genes) were collected from TCGA. The associations between CXCL13 expression levels and the stromal and immune scores and immune-related genes were analyzed by Spearman’s method. p < 0.05 and | r | >0.3 were regarded as cut-off criteria, and | r | >0.6 was considered be strongly correlated. The infiltration proportion of 22 immune cells was estimated using CIBERSORT (11), which is a tool for predicting the immunocell phenotype. We verified it using the algorithms of MCPCOUNTER, QUANTISEQ, TIMER, and XCELL. The correlation between CXCL13 expression and immune cell infiltration was analyzed using ggplot2, ggpubr and ggExtra R packages. A p value < 0.001 was used as a cut-off. Furthermore, redundancy analysis (RDA) was used to evaluate the correlation between CXCL13 and MSI, TMB, Immune scores and Stromal scores.

### Association Between CXCL13 Expression and Immunotherapeutic Response

Tumor Immune Dysfunction and Exclusion (TIDE) biomarker evaluation module was used to estimate and validate the performance of CXCL13 as a biomarker to predict patient response to immunotherapy. AUC>0.7 was the cutoff value that CXCL13 was considered to be effective for treatment response.

### Multivariate Analysis of Survival

Variables included in this part of multifactorial survival analysis included survival time, survival status, age, gender, stage, MSI, TMB, immune score, stromal score, and CXCL13 gene expression. The tumors were those that CXCL13 was correlated with OS in the previous Kaplan-Meier method and univariate cox regression analysis. We used R software and cox.zph function to test whether all the dependent variables of cox model and cox model satisfy proportional hazard (PH) assumptions. Multivariate cox regression method was used for models satisfying PH assumptions, time-dependent cox regression was used for models not satisfying PH assumptions. Covariates that do not meet the PH assumption were defined as time-dependent covariates, which were introduced into the cox regression model to form a cox regression model with time-dependent covariates. And we calculated -2 log Likelihood (-2 LL) and cordance index (c index) of the full model which included all interactions and the reduced model which contained only main effects. Bootstrap re-sampling method was used for cross-validation. Likelihood ratio test was used to test the goodness-of-fit of the models. C index was used to test the discriminating ability of the model.

### Correlation Analysis of CXCL13 Expression Levels and Mutation, Copy Number Variation, and DNMT in Pan-Cancer

The mutation data and copy number variation (CNV) data for CXCL13 were downloaded from the TCGA database. We evaluated the change frequency, mutation type, and CNA (copy number change) results for CXCL13 in all TCGA tumors using cBioPortal (https://www.cbioportal.org/) (12). The correlations between CXCL13 mutation, CNV, and CXCL13 expression levels were evaluated using the Wilcoxon and Kruskal-Wallis tests. DNA methyltransferase plays a significant part in modifying gene expression and chromatin structure. Thus, we examined the associations between CXCL13 expression and the expression of four methyltransferases (i.e., DNMT1, DNMT2, DNMT3A, and DNMT3B) using Pearson correlation analysis. The cut-off value was |r | ≥ 0.2 and p < 0.05.

### Association Between CNV of CXCL13 and Overall Survival in Pan-Cancer

We divided the samples into “deletion”,”normal” and “Gain” groups according to the types of mutation, and log-rank test was used to illustrate the correlations between the CXCL13 CNV levels and overall survivals in 33 cancer types. A p value < 0.05 was used as a cut-off.

### GSEA Analysis Across the GTEx and TCGA Datasets

The Gene Set Enrichment Analysis (GSEA) enrichment analysis was performed on 33 tumors from TCGA and 31 normal tissues from GTEx, respectively. GSEA website (https://www.gseamsigdb.org/gsea/downloads.jsp) was used to acquire Gene Ontology (GO) and Kyoto Encyclopedia of Genes and Genomes (KEGG) gene sets (c5.all.v7.1.symbols.gmt and c2.cp.kegg.v7.1.symbols.gmt) to analyze the GO and KEGG functional annotation and enrichment pathways of CXCL13 in 33 types of cancer. For each tumor or normal tissue, we divided the samples into high and low groups according to the median value of CXCL13 expression, and used using R software (version 3.6.3) *via* R-package “limma”,”org.Hs.eg.db”, “clusterProfiler” and “enrichplot” for GO and KEGG analysis.

### Quantitative Real-Time PCR (qRT-PCR)

All clinical samples used to detect CXCL13 mRNA levels were collected from the First Hospital of China Medical University, Shenyang, China. Total RNA was extracted from cancer and adjacent non-tumor tissues, including 40 cases of STAD with 25 adjacent cancer tissues, and 30 cases of READ with 30 adjacent cancer tissues. SYBR-green PCR Master Mix and Real-time PCR 480 system were applied to perform qRT-PCR. The melting curve results are all single peaks. CXCL13, PDL1, ICOS and beta-actin primer sequences were provided in **Supplementary Table S1**. Study approval was obtained from the Institute Research Medical Ethics Committee of the First Affiliated Hospital of China Medical University and all individuals provided written informed consents. 2−ΔCt was used to calculate relative expression and SPSSv25.0 (IBM, SPSS, and Chicago, IL, United States) and R software (Version 3.6.3) were utilized to perform data analysis. Paired T test was used to detect cancer tissues and paracancer tissues differences in 25 pairs of STAD and 30 pairs of READ. Spearman correlation analysis was used to analyze the association among genes of CXCL13-PDL1 and CXCL13-ICOS in 40 STAD. A p value < 0.05 was used as a cut-off.

## Results

### CXCL13 Expression Between Normal and Tumor Tissue Samples

**Figure 1:**
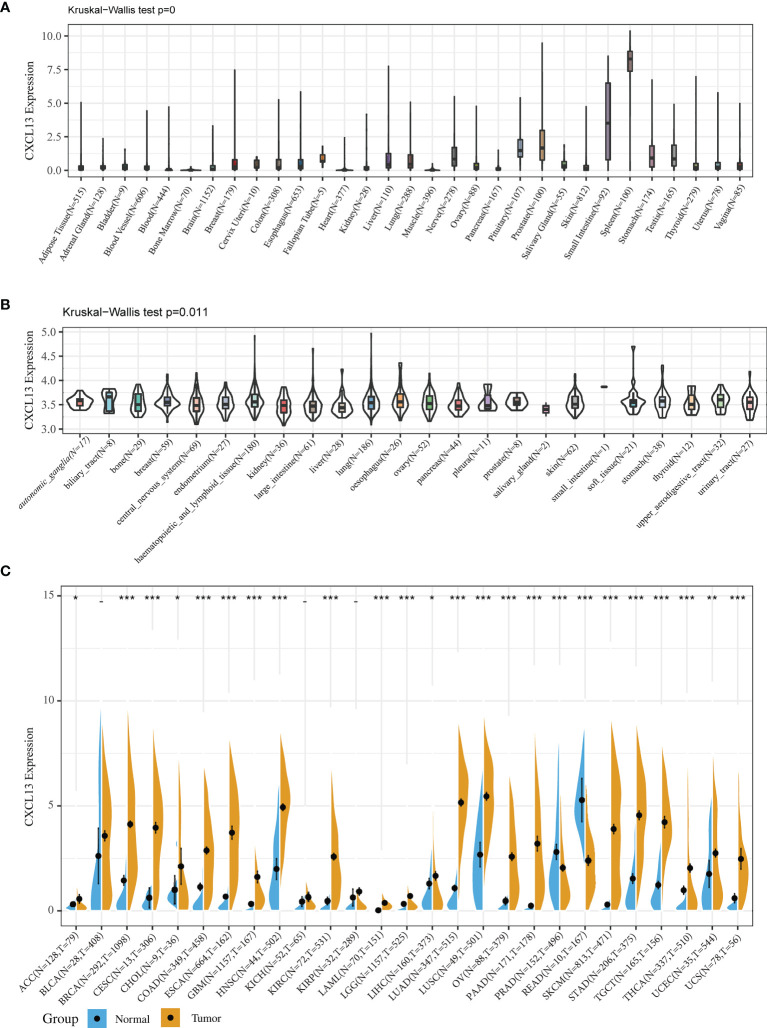
CXCL13 expression in normal tissues and tumors. **(A)** CXCL13 expression in 31 normal tissues from the GTEx database. **(B)** CXCL13 expression in 21 cancer cell lines from the CCLE database. **(C)** CXCL13 expression in 27 cancer types from the GTEx database and TCGA database. *P < 0.05, **P < 0.01, ***P < 0.001.

We investigated CXCL13 expression in 31 normal human tissue types using the GTEx dataset. CXCL13 was expressed at low levels in most normal tissue (**Figure 1A**). We also examined the CXCL13 levels in 23 cell lines using the CCLE database and found low to medium CXCL13 relative expression levels in most cell lines, consistent with the GTEx analysis results (**Figure 1B**).

Because the number of normal specimens in the TCGA database was relatively small, we analyzed differential CXCL13 expression after combining the whole TCGA dataset with the GTEx normal data. The analysis revealed significant differences in CXCL13 expression across 24 tumors, with higher CXCL13 expression levels found in 22 cancer types (ACC, BRCA, CESC, CHOL, COAD, GBM, ESCA, HNSC, KIRC, LAML, LGG, LIHC, LUAD, LUSC, OV, SKCM, STAD, PAAD, TGCT, THCA, UCEC, and UCS) and lower expression in two cancer types (PRAD and READ) compared to the normal tissues (**Figure 1C**).

**Figure 2:**
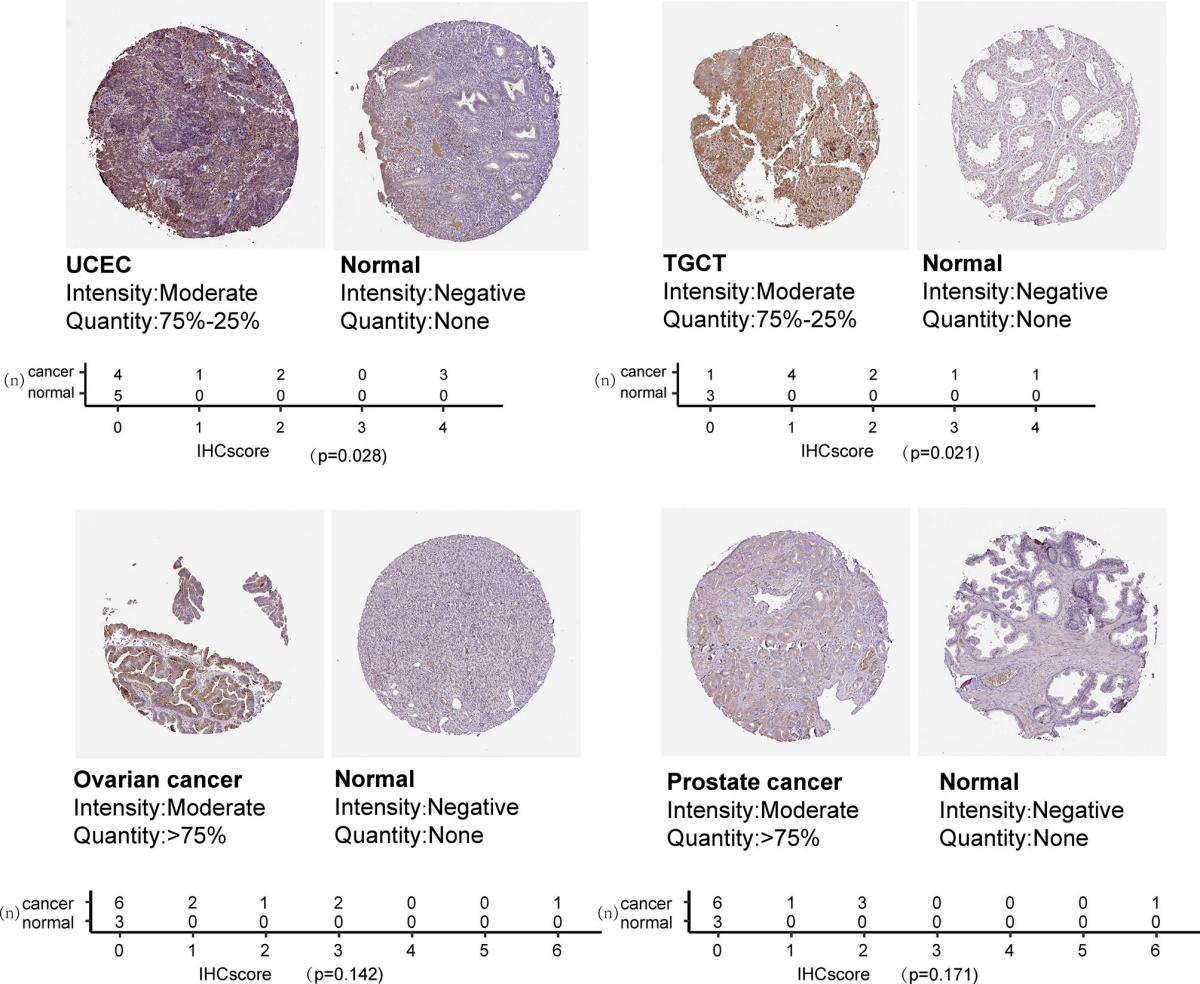
CXCL13 protein expression based on immunohistochemistry staining and the quantification of immunohistochemical staining.

We also examined CXCL13 protein levels in different cancer types using the immunohistochemistry results from ‘The Human Protein Atlas’ database. CXCL13 expression was higher in UCEC and TGCT than in the corresponding normal tissues (**Figure 2**). Immunohistochemical results showed that the protein level of CXCL13 was also consistent with our analysis.

### Correlation Between CXCL13 Expression Levels and Clinicopathological Features and Prognosis in Pan-Cancer

**Figure 3:**
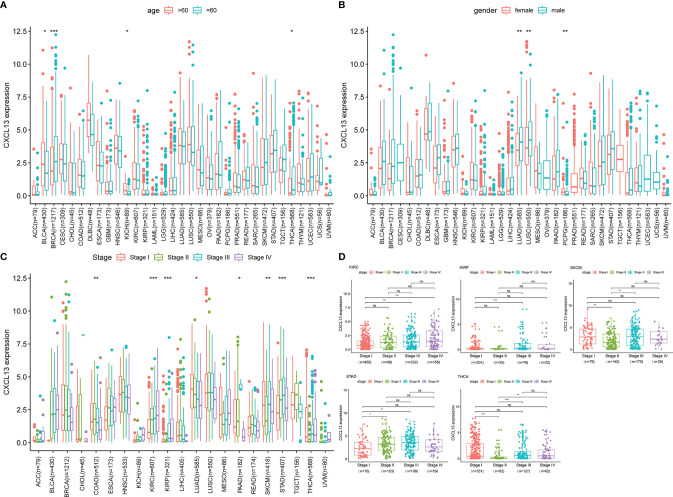
The clinical correlation of CXCL13. **(A)** represents the correlation between age and CXCL13; **(B)** shows the correlation between gender and CXCL13; **(C)** represents the correlation between tumor stage and CXCL13; **(D)** demonstrates the correlation between CXCL13 and stages between pairwise groups. *P < 0.05, **P < 0.01, ***P < 0.001. ns, no significance.

CXCL13 was differentially highly expressed among elder patients of the BLCA and KICH, whereas it was weakly expressed in BRCA and THCA (**Figure 3A**). As illustrated in **Figure 3B**, CXCL13 expression was higher among male of LUAD and higher among female of PCPG. Besides, CXCL13 expression was significantly correlated with tumor stages of some cancers, including COAD, KIRC, KIRP, LUAD, PAAD, SKCM, STAD and THCA (**Figure 3C**) and the results of pairwise comparison of clinical stages were illustrated in **Figure 3D**.

**Figure 4:**
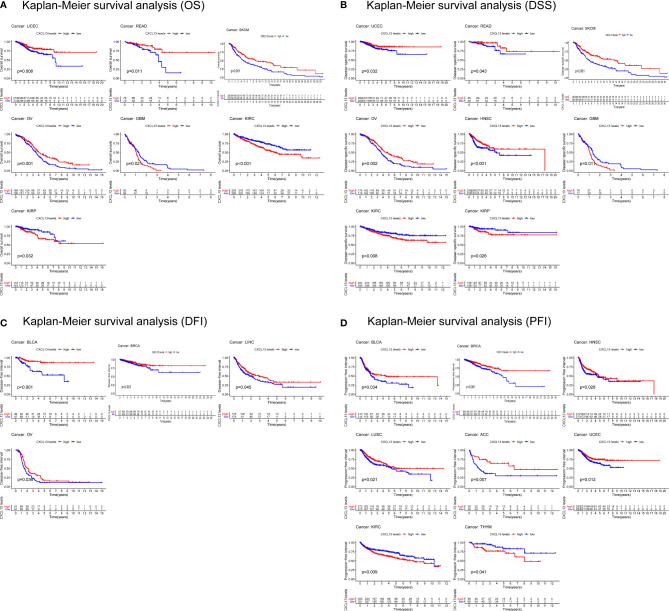
Kaplan-Meier survival curves comparing the high and low expression of CXCL13 gene in various cancer types. **(A)** OS in 7 cancer types. **(B)** DSS in 8 cancer types. **(C)** DFI in 4 cancer types. **(D)** PFI in 8 cancer types.

**Figure 5:**
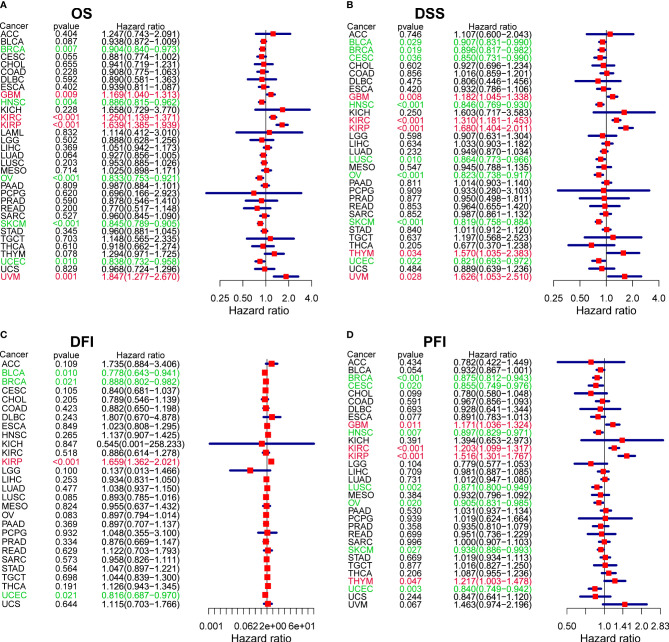
The forest plots of univariate Cox regression analyses. **(A)** OS. **(B)** DSS. **(C)** DFI. **(D)** PFI. Red items indicate CXCL13 expression is a contributing factor to mortality. Green items indicate CXCL13 expression is a factor that inhibits death.

In parallel, we investigated the role of CXCL13 in pan-cancer prognosis using the TCGA dataset. Kaplan-Meier cumulative curves showed that CXCL13 expression levels were related to OS in 7 tumors, related to DSS in 8 tumors, related to DFI in 4 tumors, related to PFI in 8 tumors (**Figures 4A** **–D**). Furthermore, we performed cox analysis to investigate CXCL13-related survival OS, DSS, DFI, and PFI and found CXCL13 expression levels were related to OS in 9tumors, related to DSS in 13 tumors, related to DFI in 4 tumors, related to PFI in 11 tumors (**Figures 5A** **–D**). Noticeably, Combining the two assays, we found that CXCL13 was associated with OS in 6 tumors (UCEC, SKCM, OV, GBM, KIRC and KIRP), associated with DSS in 7 tumors (UCEC, SKCM, OV, HNSC, GBM, KIRC and KIRP), associated with DFI in 2 tumors (BLCA and BRCA), associated with PFI in 6 tumors (BRCA, HNSC, LUSC, UCEC, KIRC and THYM). Finally, we identified CXCL13 as a protective factor in the prognosis of BRCA, UCEC, HNSC, SKCM, OV, BLCA, CESC, and LUSC, whereas it was identified as a harmful factor in the prognosis of KIRP, KIRC, GBM, THYM, and UVM.

Finally, we tested the correlation between clinical outcomes (age, gender and tumor stages) and overall survival, the results were shown in **Supplementary Table S2**. Gender was not associated with OS in all tumors, age was associated with OS in 7 tumors, and stage was associated with OS in 17 tumors. In these tumors, the median survival time decreases with age and stage.

### Correlation Between CXCL13 Expression Levels and MMRs, MSI, and TMB in Pan-Cancer

**Figure 6:**
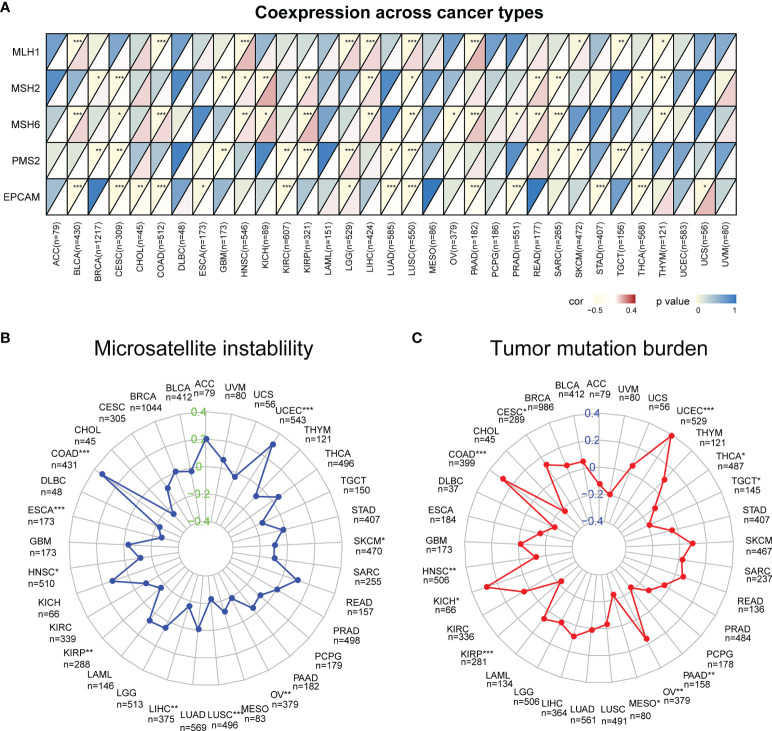
Correlation between CXCL13 expression and the mutation levels of **(A)** Mismatch Repair Genes (MMRs), **(B)** Microsatellite Instability (MSI), **(C)** Tumor Mutation Burden (TMB). *P < 0.05, **P < 0.01; ***P < 0.001.

MSI and TMB are significantly related to the sensitivity of immunotherapy, and the occurrence of MSI is closely related to the function of mismatch repair system, so we studied the correlation of CXCL13 expression with MMR genes (MLH1, MSH2, MSH6, PMS2, EPCAM), MSI and TMB. We found that CXCL13 expression levels were associated with five MMR genes (EPCAM, PMS2, MSH6, MSH2, and MLH1) in 25 cancer types (**Figure 6A**). For most tumors were the negative correlation except BLCA, HNSC, KICH, KIRP, LGG, LIHC, PAAD and READ. The association between CXCL13 and MSI was significant in 9 cancer types. Specifically, positive associations were found in UCEC and COAD, while negative correlations were observed in ESCA, HNSC, KIRP, LUSC, LIHC, OV and SKCM (**Figure 6B**). The results also suggested a correlation between CXCL13 expression levels and TMB in 11 cancer types. CXCL13 expression levels were positively related to TMB in 5 cancer types (UCEC, KICH, OV, CESC, and COAD) but negatively related to TMB in 6 different cancer types, including HNSC, TGCT, KIRP, PAAD, MESO and THCA (**Figure 6C**).

### Correlation Between CXCL13 Expression and Tumor Immune Microenvironment Environment (TIME) in Pan-Cancer

**Figure 7:**
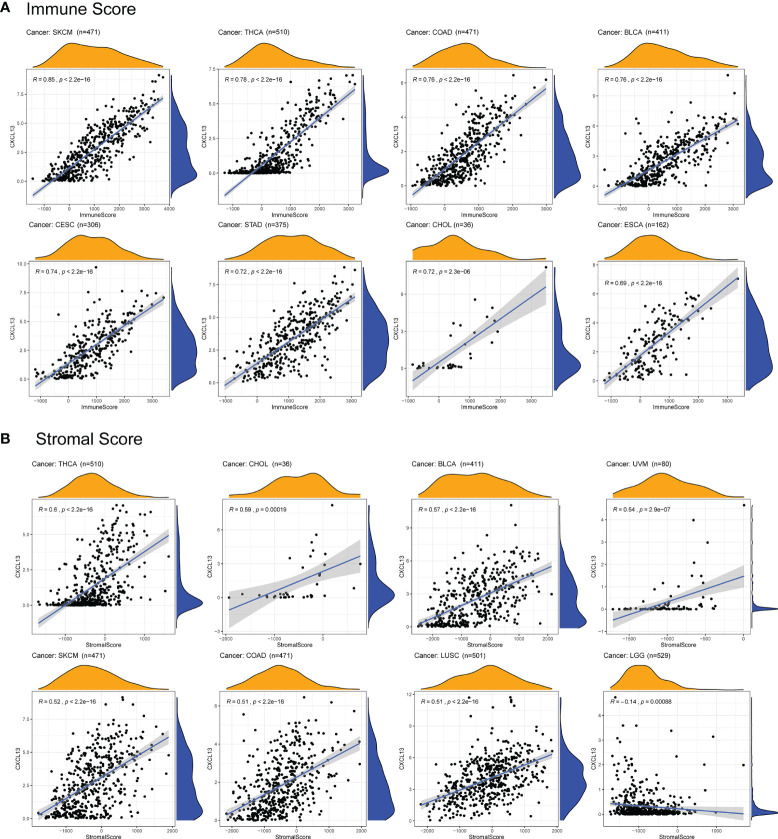
Correlation between CXCL13 expression and immune scores and stromal scores by ESTIMATE algorithm. **(A)** The top eight tumors most strongly associated with the immune scores. **(B)** The top eight tumors most strongly associated with the stromal scores.

**Figure 8:**
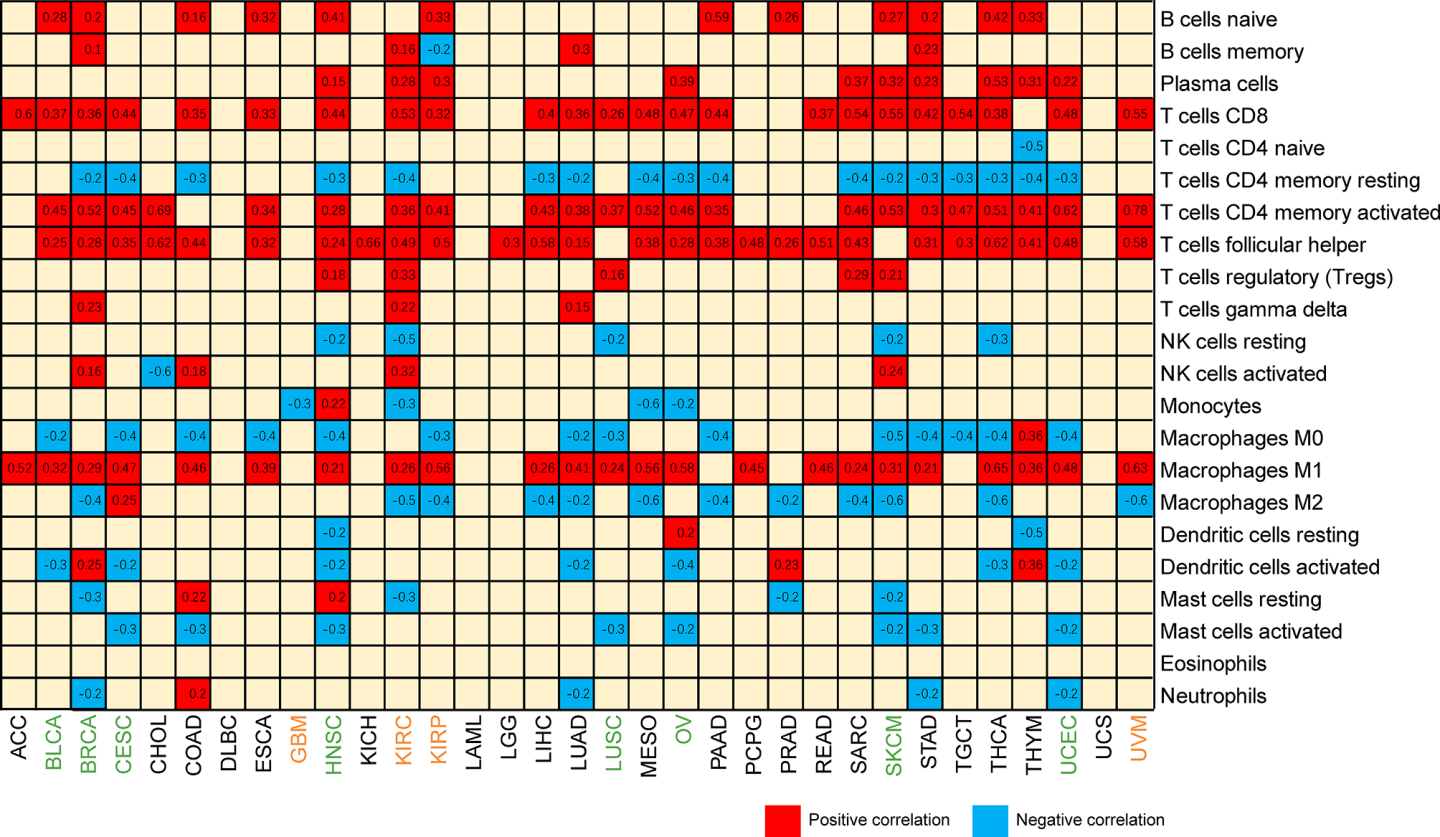
Heatmap of the correlation between CXCL13 expression and infiltrating levels of immune cells. Blue represents negative correlation, and red represents positive correlation. Both red and blue represent P<0.001.

With the development of omics technology, we gradually understand the complexity and diversity of TIME and its important influence on immunotherapy. Therefore, it is of great significance to explore the relationship between CXCL13 and TIME. We evaluated the correlations between CXCL13 expression and stromal scores, immune scores and immune cell infiltration. The results indicated that CXCL13 expression levels were positively associated with the immune scores in 21 cancers as well as the stromal scores in 14 cancer types except LGG (p < 0.05 and |R| > 0.3). The top eight tumors most strongly associated with the two scores were shown in the **Figure 7**. The remaining analysis results were shown in **Supplementary Figure 1**. CXCL13 expression levels were significantly correlated with the levels of many infiltrating immune cells (**Supplementary Figure 2**), especially CD8+T cells, M1 macrophages and follicular helper T cells based on CIBERSORT algorithm (**Figure 8**). We further used algorithms of TIMER, QUANTISEQ, XCELL and MCPCOUNTER to validate the results, and the results were highly consistent (**Supplementary Figure 3A**).

**Figure 9:**
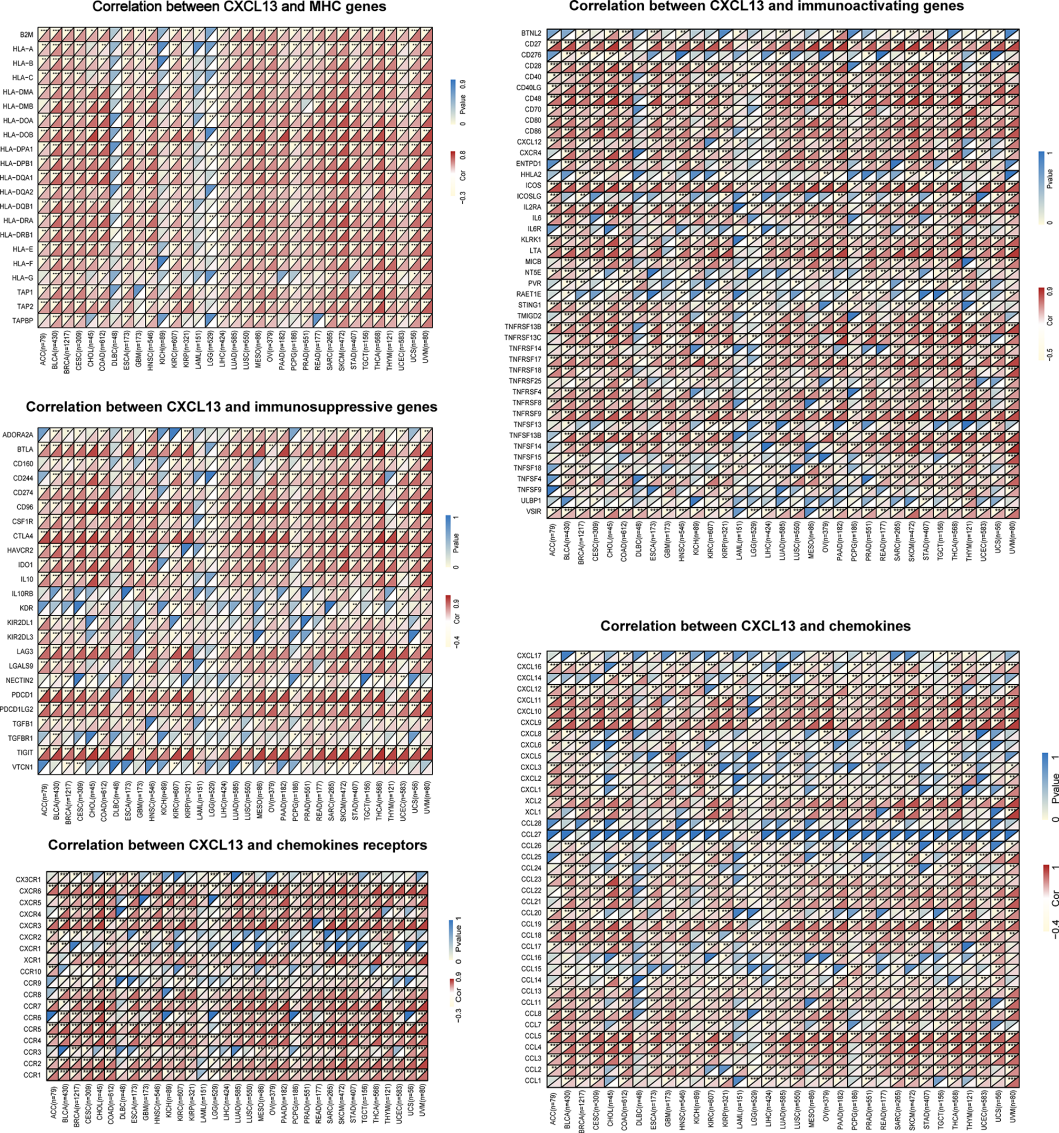
The correlation between CXCL13 and immune-related genes. *P < 0.05, **P < 0.01, ***P < 0.001.

Next, we explored the relationships of immune-related genes (chemokine, chemokine receptors, MHC, immunosuppressive genes, immunoactivating genes) and CXCL13 expression by gene co-expression analyses in 33 tumors. The results indicated that immunosuppressive genes including TIGIT, PDCD1(PD1), LAG3, CTLA4, CD96, CD274(PDL1), BTLA and immunoactivating genes including CD27, CD48, ICOS, LTA, TNFRSF9, TNFSF13B and chemokines including CXCL9, CXCL10, CXCL11, CCL19, CCL5 were strongly correlated with CXCL13 gene expression (p<0.05, R>0.6) in majority cancers (**Figure 9**).

Furthermore, we used redundancy analysis to evaluate the correlation between CXCL13 and MSI, TMB, Immune scores and Stromal scores. The results showed that CXCL13 was still positively correlated with TMB in CESC, COAD, HNSC, KICH, OV, UCEC, and negatively correlated with KIRP, MESO and THCA. And CXCL13 was still positively correlated with MSI in COAD and SKCM and negatively in HNSC and KIRP. In the previous correlation analysis, CXCL13 was positively correlated with immunescore in 21 tumors, and was still positively correlated with immunescore in 17 of the 21 tumors analyzed by RDA. Meanwhile, CXCL13 was positively correlated with stromalscore in 14 tumors in the previous correlation analysis, and was still positively correlated with stromalscore in 12 of the 14 tumors analyzed by RDA (**Supplementary Figure 3B**). The results of RDA analysis were highly consistent with those of spearman method.

### Association Between CXCL13 Expression and Immunotherapeutic Response

**Figure 10:**
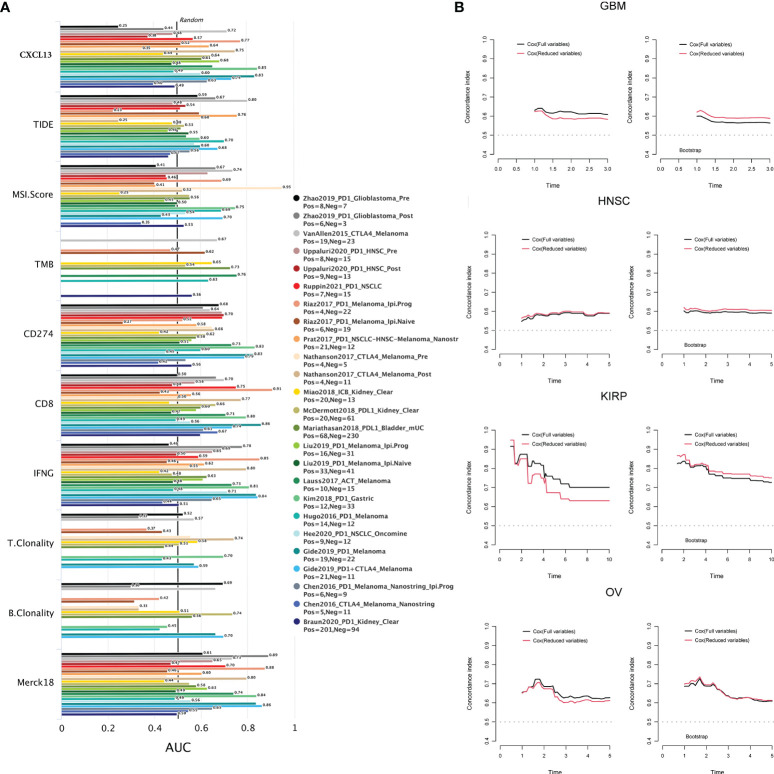
**(A)** The correlation between CXCL13 and the immunotherapeutic response. **(B)** The c index and the results of the cross-validation by bootstrap re-sampling method of the full model and reduced model in GBM, HNSC, KIRP and OV.

What’s more, we used TIDE biomarker evaluation module to estimate and validate the performance of the comparison between CXCL13 and other published biomarkers based on their predictive power of response outcome. CXCL13 performed well (AUC>0.7) in the diagnosis of immunotherapy response in 6 cohort studies (**Figure 10A**). Among them, in the studies of “Kim2018 PD1 Gastric” (AUC=0.85) and “Gide2019 PD1 Melanoma” (AUC=0.83), the CXCL13 predictive power of response outcome was better than other published biomarkers.

### Multivariate Analysis of Survival

The results of multifactorial analysis showed that CXCL13 was still associated with prognosis in full and reduced models in KIRP, GBM, HNSC, and OV. CXCL13 was a risk factor for the prognosis of KIRP and GBM patients, while CXCL13 was a protective factor for the prognosis of HNSC and OV patients **(** **Supplementary Table S3** **)**. The results of likelihood ratio test showed that the possibility of fitting the full modes and the reduced models were equal, and the constraint conditions were valid in KIRP, GBM, HNSC, and OV **(** **Supplementary Table S3** **)**. As illustrated in **Figure 10B**, the discriminatory power of the full model was slightly better than the reduced model in GBM, KIRP and OV. But according to the results of the cross-validation by bootstrap re-sampling method, the discriminatory power of the reduced model was slightly better than the full model in GBM, HNSC and KIRP, the discriminant power of the reduced model was approximately equal to that of the full model in OV.

### Correlation Between CXCL13 Expression Levels and CXCL13 Mutation, CNV, and DNMT in Pan-Cancer

**Figure 11:**
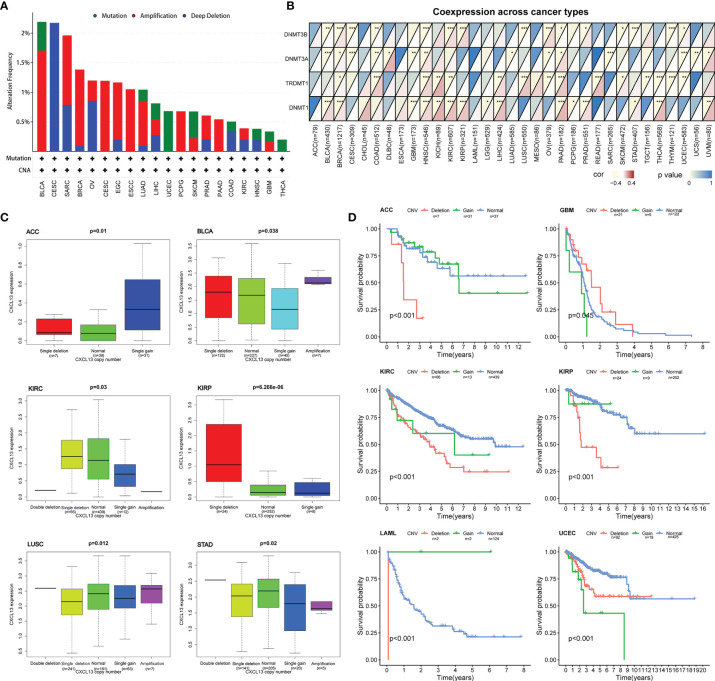
Correlation between CXCL13 expression levels and CXCL13 mutation, CNV, and DNA methyltransferase. **(A)** The mutation features of CXCL13 for the TCGA tumors using the cBioPortal database. **(B)** The correlation between CXCL13 expression level and DNA methyltransferase. **(C)** The correlation between CXCL13 expression level and CXCL13 CNV. **(D)** The correlation between CXCL13 CNV and overall survival. *P < 0.05, **P < 0.01, ***P < 0.001.

To explore whether CXCL13 gene expression was affected by its own alterations, we evaluated the correlations between CXCL13 gene mutation, CNV, and CXCL13 gene expression. We first investigated CXCL13 gene mutations using the cBioPortal database (**Figure 11A**). We assessed the correlation between the expression levels of CXCL13 and four DNA methyltransferases. A close association was observed between CXCL13 and DNMT expression in 15 cancer types (**Figure 11B**). We found that CXCL13 gene mutations did not affect its expression in any tumor type evaluated. However, correlation analysis between CXCL13 CNV and expression demonstrated that CXCL13 expression was correlated with CNV in ACC, BLCA, KIRC, KIRP, LUSC, and STAD (**Figure 11C**). Furthermore, we explored the correlation between CNV of CXCL13 and the overall survival of cancer patients. As illustrated in **Figure 11D**, the CNV of CXCL13 was correlated with the overall survival of ACC, GBM, KIRC, KIRP, LAML and UCEC.

### Function Annotation and Pathway Analysis of CXCL13 in Normal Tissues and Pan-Cancer

GSEA results of normal tissues from GTEx showed that CXCL13 was associated with metabolic functions and pathways. Such as pathways of oxidative phosphorylation, glycerophospholipid metabolism, porphyrin and chlorophyll metabolism and starch, sucrose metabolism (**Supplementary Figure 4**), and biology of gene silencing, mRNA binding, leukocyte migration, T cell activation, immune response regulating cell surface receptor, regulation of immune effector process, etc. (**Supplementary Figure 5**).

In the GSEA results of cancers from TCGA, KEGG pathway analysis showed that CXCL13 was primarily correlated with some pathways including cytokine-cytokine receptor interactions, the T-cell receptor, chemokine, JAK-STAT, natural killer (NK) cell-mediated cytotoxicity, B-cell receptor signaling pathways, primary immunodeficiency, cell adhesion molecules (CAMs), and autophagy regulation, etc. In cancers that CXCL13 harbor favorable prognosis, KEGG results suggested that “T cell receptor signaling pathway” and “NK cell mediated cytotoxicity” might be involved in the effect of CXCL13 on anti-tumor pathogenesis. Go enrichment analysis indicated that most of these genes were linked to the pathways or immune biology of T cell and NK cell, such as T cell receptor complex, T cell differentiation involved in immune response, NK cell chemotaxis, positive regulation of NK cell mediated immunity and so on. Oppositely, in cancers that CXCL13 harbor unfavorable prognosis, CXCL13 may promote tumors through chemokine signaling pathway, cytokine-cytokine receptor interaction and JAK-STAT signaling. All results of GSEA analysis were illustrated in **Supplementary Figures 6**, **7**.

### QRT-PCR Validations *In Vivo*

**Figure 12:**
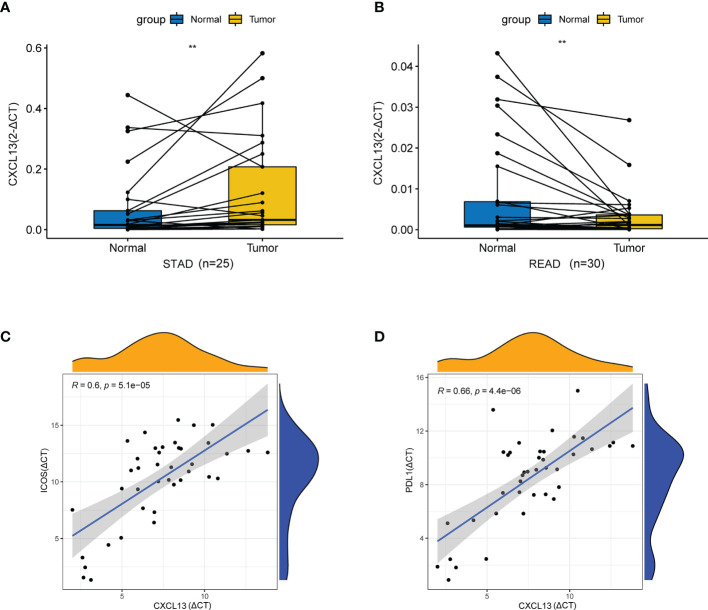
qRT-PCR validation *in vivo*. **(A)** The mRNA relative expression of CXCL13 detected by qRT-PCR in STAD and adjacent nontumor tissues. **(B)** The mRNA relative expression of CXCL13 detected by qRT-PCR in READ and adjacent nontumor tissues. **(C)** The correlation between CXCL13 and ICOS detected by qRT-PCR. **(D)** The correlation between CXCL13 and PDL1 detected by qRT-PCR. **P < 0.01.

Then, we assessed the expression of CXCL13 in STAD and READ by qRT-PCR. As illustrated in **Figure 12A**, the results showed that CXCL13 mRNA expression was higher than adjacent tissues in STAD (p < 0.01). As illustrated in **Figure 12B**, CXCL13 mRNA expression in cancer tissue was lower than adjacent tissues in READ (p < 0.01). The results were consistent with our analysis by TCGA. We also validated the results with qRT-PCR of the significant correlation in CXCL13-ICOS (r=0.6, p < 0.001), CXCL13-PDL1 (r=0.66, p < 0.001) in STAD tissues (**Figures 12C, D** **)**.

## Discussion

The present study used multilevel data to elucidate the CXCL13 expression-based panoramic picture in human cancer to clarify its likely role in cancer development, progression, and immunity. By elaboratively studying the CXCL13 transcriptional data from the TCGA, GTEx, CCLE and HPA databases, we created a portrait of the CXCL13 expression landscape in various cancers and investigated the correlations of CXCL13 expression with clinical features, prognosis, MMRs, MSI, TMB, TIME, immunotherapeutic response, CNV, DNMT, function annotation and related pathways. QRT-PCR was used to validated the cancer tissues and paracancer tissues differences of CXCL13 expression in STAD and COAD and the correlation between CXCL13 and immune genes in STAD.

The expression profile analysis revealed that, CXCL13 was highly expressed in 21 tumors among the 24 tumors with differential CXCL13 expression. According to the literature search results, higher CXCL13 expression was found in colorectal cancer (13), renal cell carcinoma (14), hepatocellular carcinoma (15), gastric cancer (16), breast cancer (17, 18), lung cancer (19), pancreatic adenocarcinoma (20), cutaneous melanoma (21) and leukemia (22), which were consistent with our study. Our study is the first to demonstrate that high CXCL13 expression occurred in CESC, CHOL, ESCA, GBM, HNSC, LGG, PAAD, UCS, UCEC, THCA, TGCT and low expression in READ.

Pan-cancer prognostic analyses indicated that CXCL13 was associated with better survival in BLCA, BRCA, HNSC, LUSC, OV, SKCM, and UCEC but a worse prognosis for GBM, KIRC, KIRP, and THYM by using Kaplan-Meier method and univariate cox regression analysis. Previous studies have shown that CXCL13 was expressed in cutaneous melanoma more than in normal tissue and was associated with better overall survival (21). CXCL13 was related to longer OS in ovarian cancer with wild-type TP53 (23). In contrast, CXCL13 was significantly upregulated in clear cell renal cell carcinoma, and high CXCL13 expression was associated with a poor prognosis (14). Our results are consistent with these findings. By constructing and testing multivariate cox regression models including full model and reduced model, it was found that CXCL13 was correlated with the prognosis of KIRP, GBM, HNSC and OV, and could be used as one of the main covariables of reduced model for these four tumors. The goodness-of-fit and discriminatory power of the reduced models were not significantly different from that of the full models with all variables. And the reduced models were more concise. These results suggested that CXCL13 may be an important prognostic marker of KIRP, GBM, HNSC and OV.

As the key mediator of tumor progression and treatment outcome, tumor microenvironment plays an important role in clinical survival and response to immunotherapy (24). TIME is a focus of cancer research and is comprised of tumor-infiltrating lymphocytes (TILs; B and T cells) and other immune cells (macrophages, neutrophils, and dendritic cells) (25). In the present study, CXCL13 levels were significantly associated with the infiltration levels of CD8+ T cells and macrophages. In cancers that CXCL13 harbor favorable prognosis, one study reported that CXCL13 was associated with better overall survival and positively correlated with infiltration of six immune cells (B cell, CD8+T cells, CD4+T cells, macrophages, neutrophils, dendritic cells) in SKCM (21). Another study showed that the higher infiltration of CD8+ T cells and macrophages were identified in the group with higher expression of immune checkpoint molecules (CD-274 (PD-L1), CTLA-4), and T cell exhaustion genes (HAVCR2, TIGIT, LAG3, PDCD1, CXCL13, and LYN) in LUSC (26), which was also consistent with our findings related to immunity genes. In cancers that CXCL13 harbor unfavorable prognosis, such as KIRC, intratumoral CXCL13 + CD8 + T cell infiltration determines poor clinical outcomes and immunoevasive contexture (27).

Furthermore, our analysis showed that CXCL13 expression levels were positively related to immune-related genes in most cancers. Among these genes, immunoactivating genes ICOS, CD27 and immunosuppressive genes CD274(PD-L1), TIGIT, LAG3, CTLA4 and BTLA have attracted our attention, which were strongly correlated with CXCL13. ICOS and its ligand are the only costimulatory molecules that mainly induce the immune response of Th2. CD27 can promote the activation of CD8+ T cells and macrophages. CD274(PD-L1), TIGIT, LAG3, CTLA4 and BTLA are all highly expressed on T cells and are associated with T cell dysfunction. Pankaj et al. found higher expression of the immune checkpoint molecules CD-274 (PD-L1) and CTLA-4, and the T-cell depletion genes (TIGIT, LAG3, PDCD1, CXCL13, and Lyn) in the high-risk group of lung cancer, which may be more suitable for PD-L1 blocking or other checkpoint blocking immunotherapies (26). Li et al. identified dysfunction-related cell modules (TIGIT, PD-1, LAG3 and CXCL13) by gene co-expression analysis in CD8+T cell metacells (28). Our qRT-PCR results *in vivo* also verified the significant correlation in CXCL13-ICOS (r=0.6, p < 0.001), CXCL13-PDL1 (r=0.66, p < 0.001) in STAD tissues.

TMB and MSI are important factors influencing the efficacy of immune checkpoint inhibitors in some cancers (29–31). Higher TMB levels and MSI-H are indicative of a better response to immune checkpoint inhibitors (29, 32). TMB is also a potential independent biomarker for MSI-high metastatic colorectal cancer and can stratify patients according to their immune checkpoint inhibitor responses (33). MMRs system ensure the stability of DNA replication under normal conditions and defects in the MMR genes could result in MSI. Hence, we explored the relationship between CXCL13 expression and MMRs, MSI, and TMB. The results of Spearman and RDA analysis confirmed that CXCL13 expression levels were positively correlated with both MSI and TMB in COAD and negatively in KIRP. This indicated that CXCL13 might have an indirect effect on the immunotherapeutic response of KIRP and COAD. Subsequently, we used TIDE module to investigate the correlation between CXCL13 and the immunotherapeutic response. CXCL13 performed well (AUC>0.7) in the diagnosis of immunotherapy response in 6 cohort studies. Two cohort studies used PD1 to treat melanoma, two cohort studies used CTLA4 to treat melanoma, one cohort study combined PD1 with CTLA4 to treat melanoma, and one cohort study used PD1 to treat gastric cancer. Presently, there are few studies on the correlation between CXCL13 and MSI or immunotherapy. Zhang et al. found that only CXCL13+BHLHE40+ Th1-like cells were selectively enriched in patients with microsatellite-unstable colorectal tumors, which might explain their favorable response to immune checkpoint blockade by using single-cell sequencing (8).

Somatic cell gene mutations and germline cell polymorphisms are typically the basic variations in DNA sequences, and the key variations of gene structure usually result in gene expression changes. In addition, DNA methylation is a critical epigenetic modification that can also regulate gene expression levels. Our study analyzed CXCL13 mutations and CNV and DNA methyltransferases and their correlations with CXCL13 expression levels to better understand the importance of intrinsic mechanisms for regulating CXCL13 expression. Our findings indicated no association between CXCL13 expression levels and its mutations; however, additional studies are required to verify these results. We observed both CXCL13 copy number increases and decreases in ACC, LUSC, and STAD overexpressing CXCL13. Previous studies demonstrated that upregulated copy numbers are often associated with upregulated gene expression. However, complicated regulatory mechanisms that weaken or reverse the association between gene expression and CNV can also exist (34). Transcriptional and post-transcriptional regulation may occur, leading to inconsistent changes in expression and copy number. The mechanisms underlying the suppression of gene expression require further study. Changes in DNA methylation play a crucial role in oncogenesis. In this study, CXCL13 expression levels were closely related to the expression levels of the four DNA methyltransferases DNMT3B, DNMT3A, DNMT2, and DNMT1) in human tumors, particularly in HNSC, LUSC, KIRC, and KICH. Meanwhile, we have explored the correlation between CNV of CXCL13 and the overall survival of cancer patients. We found that CXCL13 CNV affected clinical outcomes in 6 types of cancer. In short, genetic changes play an important part in regulating CXCL13 expression and prognosis of cancer patients, the regulation mechanisms deserve more comprehensive investigation.

By GSEA enrichment analysis of normal and tumor tissues, we found that CXCL13 functions and pathways in normal tissues may be mainly related to metabolism and some basic immune functions, while in tumor tissues, CXCL13 was related to tumor-related pathways and functions. CXCL13 may play an anti-tumor and prognostic protective role through T cell and NK cell mediated pathway and cytotoxicity. CXCL13 may promote the occurrence, development and poor prognosis of cancer by activating other oncogenic pathways such as JAK-STAT and NF-KB pathways) through chemokine pathway or cytokine receptor pathway. In addition to that, CXCL13 has a crucial function in B-cell terminal differentiation and the development of B- and T-cell-mediated immune responses (35, 36). By promoting antigen presentation, CXCL13 ensures that naive B cells are involved in promoting immune responses (37). Non-lymphoid organs with aberrant CXCL13 overexpression can promote the development of TLSs. Rita et al. observed that CD8+ CD20+ tumors formed TLSs, which could help predict clinical outcomes and which patients should receive immune checkpoint blockade therapy (38).

In conclusion, this study comprehensively revealed the expression profile for CXCL13 in human cancers, explored the correlation between CXCL13 expression levels and clinicopathological feature and prognosis, tumor cell biological characteristics, and tumor immunity. It also clarified the influence of genetic variations in CXCL13 structure on CXCL13 expression and identified the functional pathways that involve CXCL13. Thus, our research provides an accurate and detailed reference to better understand the role of CXCL13 in tumor genesis, progression and prognosis, as well as the role of future immunotherapy. Based on these findings, CXCL13 might be a useful biomarker for determining the diagnosis and prognosis of human cancers and also a biomarker for evaluating the efficacy of immunotherapy. The limitations of this study are that the relevant functions and mechanisms of CXCL13 need to be further verified by further experiments.

## Data Availability Statement

The original contributions presented in the study are included in the article/**Supplementary Material**. Further inquiries can be directed to the corresponding author.

## Ethics Statement

The studies involving human participants were reviewed and approved by the Institute Research Medical Ethics Committee of the First Affiliated Hospital of China Medical University. The patients/participants provided their written informed consent to participate in this study.

## Author Contributions

YY conceived the study. HZ and YY drafted the manuscript and performed the analysis. HZ and HY performed the literature search and collected the data. HZ and JC contributed to drafting the manuscript and interpreting data. All authors read and approved the final manuscript.

## Funding

This study was supported by The National Key R&D Program of China (2018YFC1311600)

## Conflict of Interest

The authors declare that the research was conducted in the absence of any commercial or financial relationships that could be construed as a potential conflict of interest.

## Publisher’s Note

All claims expressed in this article are solely those of the authors and do not necessarily represent those of their affiliated organizations, or those of the publisher, the editors and the reviewers. Any product that may be evaluated in this article, or claim that may be made by its manufacturer, is not guaranteed or endorsed by the publisher.

## Supplementary Material

Supplementary Figure 1 The correlation between CXCL13 expression and immune scores and stromal scores.

Supplementary Figure 2 The correlation between CXCL13 expression and 22 kinds of immune cell infiltration. The immune cell infiltration was calculated by CIBERSORT algorithm.

Supplementary Figure 3
**(A)** The correlation between CXCL13 expression and immune infiltration of CD8+ T cell and macrophage by the algorithms of MCPCOUNTER, QUANTISEQ, TIMER, and XCELL. **(B)** The results of RDA analysis.

Supplementary Figure 4 KEGG pathway analysis of CXCL13 by GSEA analysis based on the GTEx.

Supplementary Figure 5 GO functional annotation of CXCL13 by GSEA analysis based on the GTEx.

Supplementary Figure 6 KEGG pathway analysis of CXCL13 by GSEA analysis based on the TCGA.

Supplementary Figure 7 GO functional annotation of CXCL13 by GSEA analysis based on the TCGA.
