# Supplementary figures and images for: An Integrated Pan-Cancer Analysis of 33 Human Cancers Reveals the Potential Clinical Implications and Immunotherapeutic Value of C-X-C Motif Chemokine Ligand 13
Source: Front Oncol. 2022 Jan 24;12:791962. doi: 10.3389/fonc.2022.791962 (PMC8818761; doi:10.3389/fonc.2022.791962)

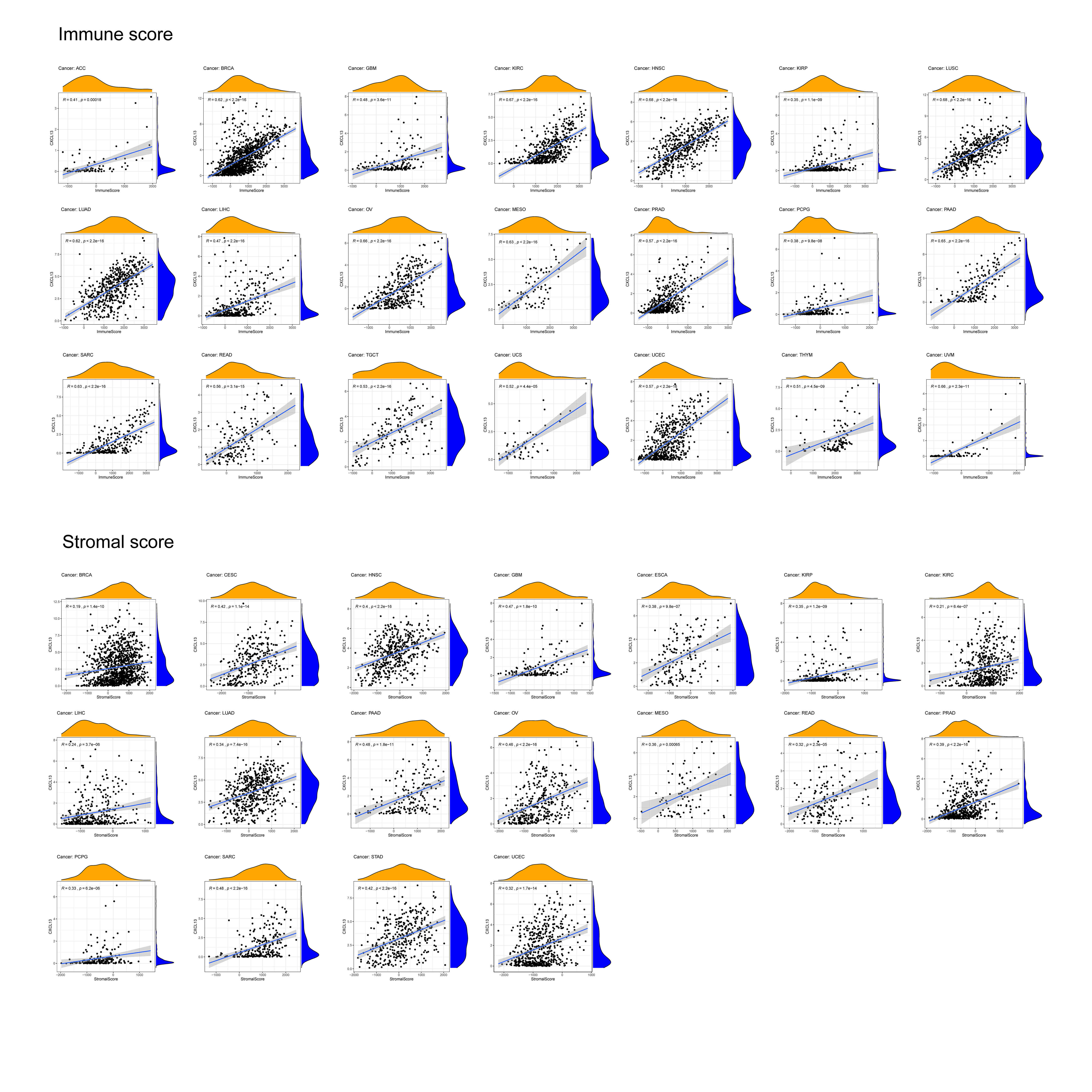

Supplement: Supplementary Figure 1 — The correlation between CXCL13 expression and immune scores and stromal scores. [file Image_1.tif]

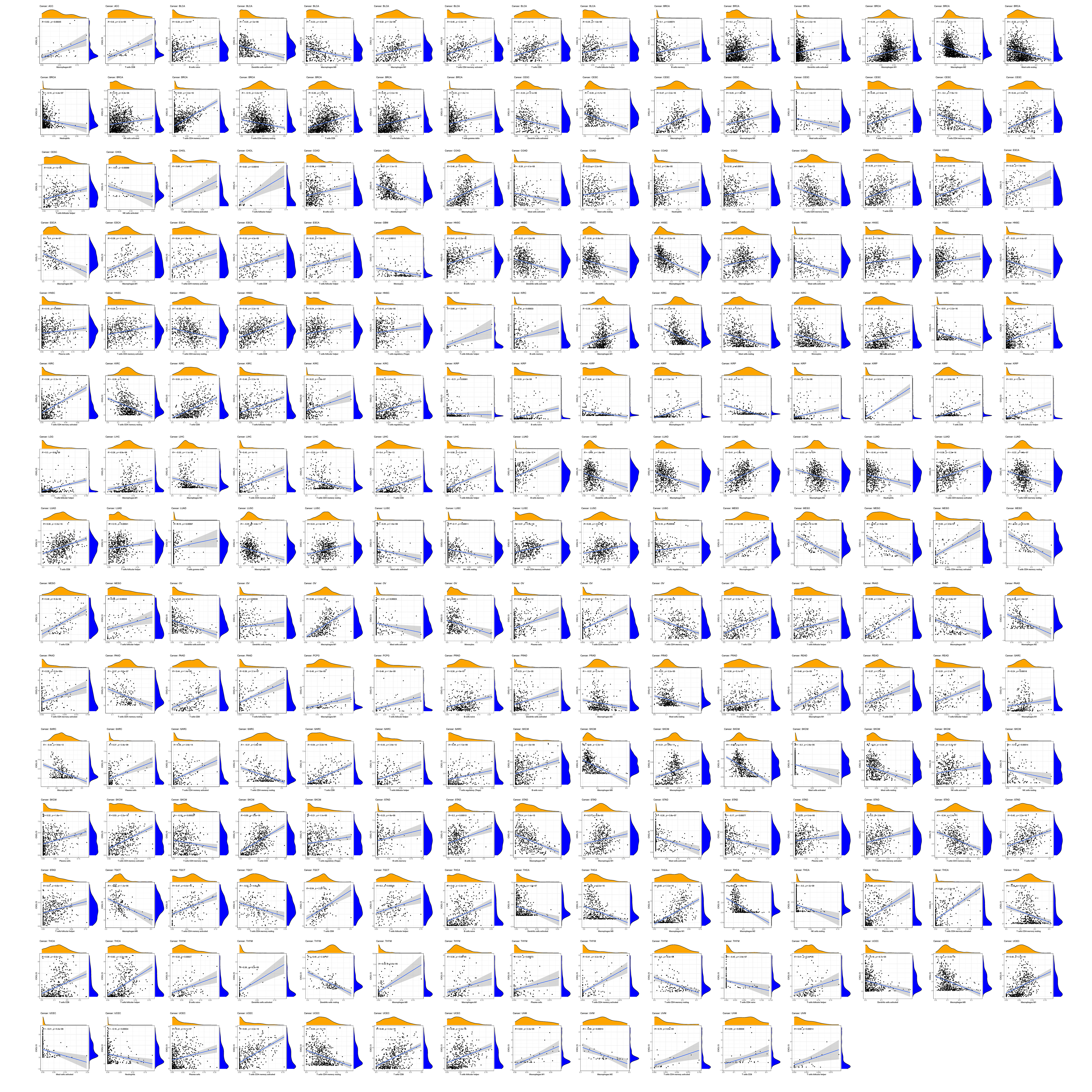

Supplement: Supplementary Figure 2 — The correlation between CXCL13 expression and 22 kinds of immune cell infiltration. The immune cell infiltration was calculated by CIBERSORT algorithm. [file Image_2.tif]

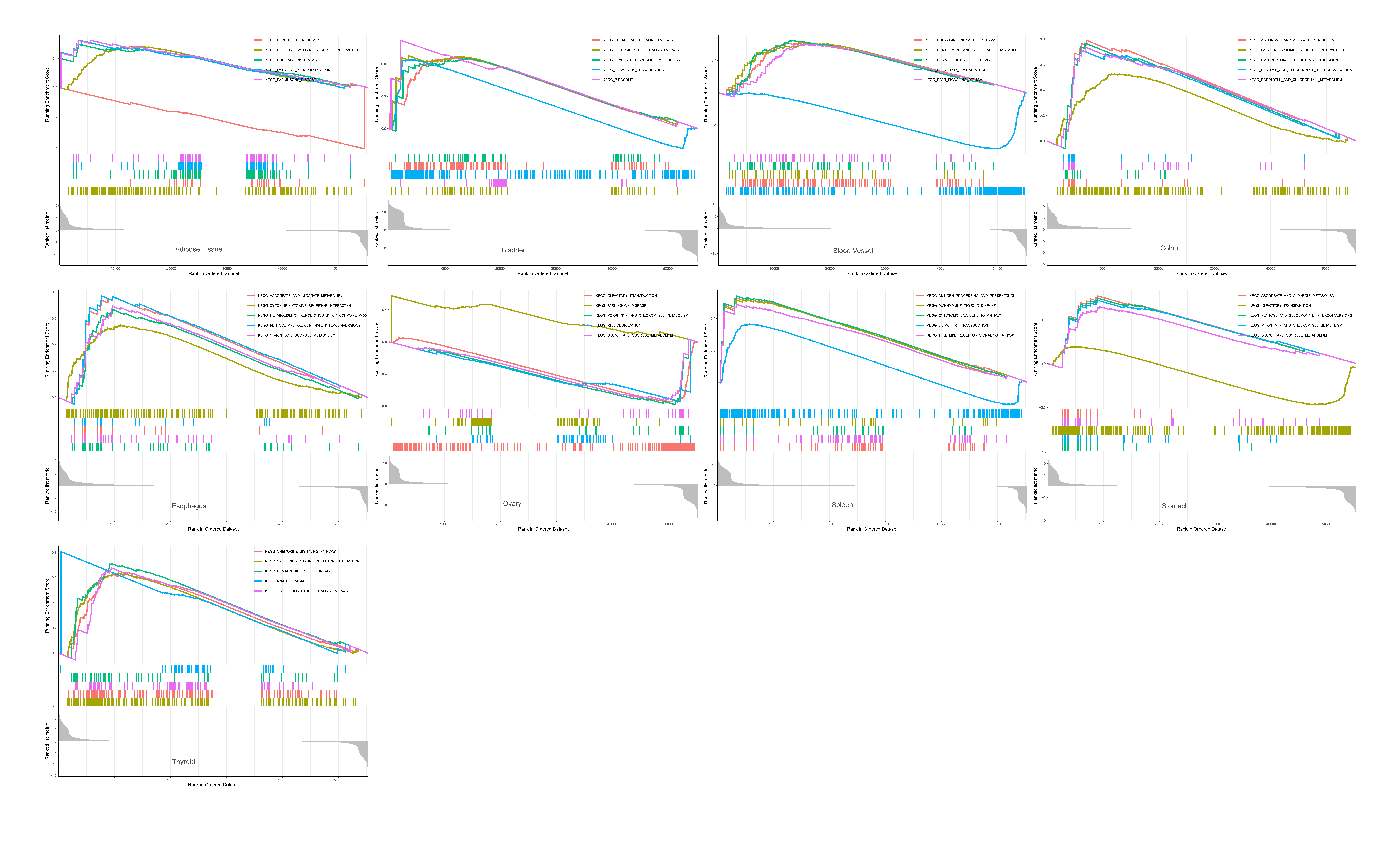

Supplement: Supplementary Figure 4 — KEGG pathway analysis of CXCL13 by GSEA analysis based on the GTEx. [file Image_4.tiff]

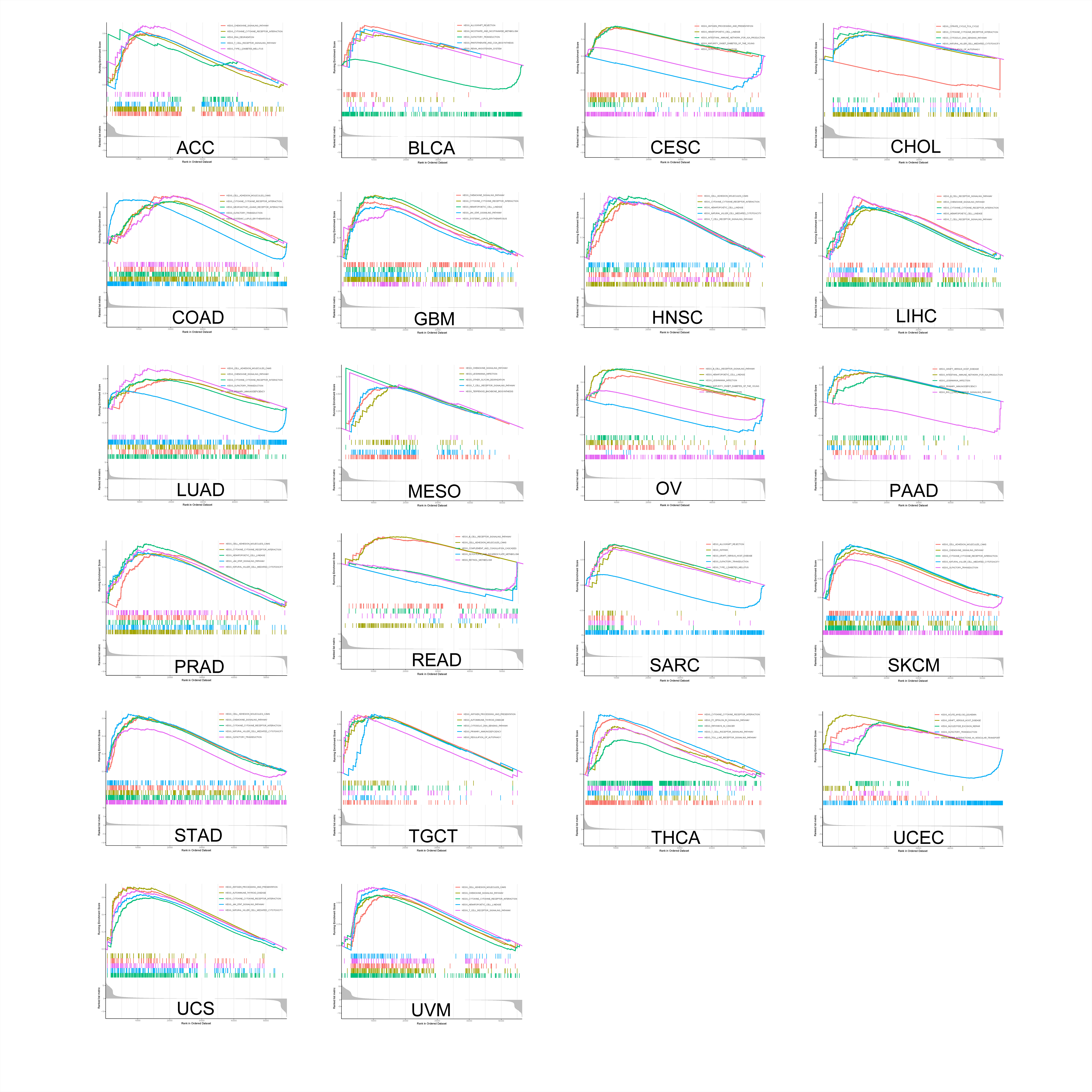

Supplement: Supplementary Figure 6 — KEGG pathway analysis of CXCL13 by GSEA analysis based on the TCGA. [file Image_6.tif]
